# Seroprevalence of SARS-CoV-2 in Sweden, April 26 to May 9, 2021

**DOI:** 10.1038/s41598-022-15183-w

**Published:** 2022-06-25

**Authors:** Jessica Beser, Ilias Galanis, Theresa Enkirch, Sharon Kühlmann Berenzon, Edward van Straten, Jan Duracz, Marie Rapp, Katherina Zakikhany, Mikael Mansjö, Julia Wigren Byström, Mattias N. E. Forsell, Ramona Groenheit, Karin Tegmark Wisell, Andreas Bråve

**Affiliations:** 1 Public Health Agency of Sweden, Solna, Sweden; 2 Department of Clinical Microbiology, Umeå University, Umeå, Sweden; 3 European Centre for Disease Prevention and Control (ECDC), European Public Health Microbiology Training Programme (EUPHEM), Solna, Sweden

**Keywords:** Immunology, Microbiology, Diseases

## Abstract

A national point seroprevalence study of SARS-CoV-2 was conducted in Sweden in April–May 2021. In total, 2860 individuals 3 to 90 years old from a probability-based web panel were included. Results showed that an estimated 32.6% of the population in Sweden had detectable levels of antibodies, and among non-vaccinated 20.1% had detectable levels of antibodies. We tested for differences in seroprevalence between age groups and by sex and estimated seroprevalence among previously infected participants by time since reporting.

## Introduction

At the beginning of April 2021, Sweden experienced a third wave of COVID-19, SARS-CoV-2 lineage B.1.1.7 (Alpha) variant dominating^1^. Vaccination against COVID-19 started in December 2020 with the age group 70 years and older being one of the prioritized groups, extending to include the 65 years and older in beginning of March 2021. By April 2021, 23% of individuals over 65 years and 8% of the Swedish population as a whole were fully vaccinated by either of Comirnaty, Spikevax or Vaxzevria vaccines^2^. In this report, we present the results of a point seroprevalence study of SARS-CoV-2 in April/May 2021 that was conducted in order to estimate seroprevalence at a national level at this time point.

### Study participants and serological analyses

**Figure 1:**
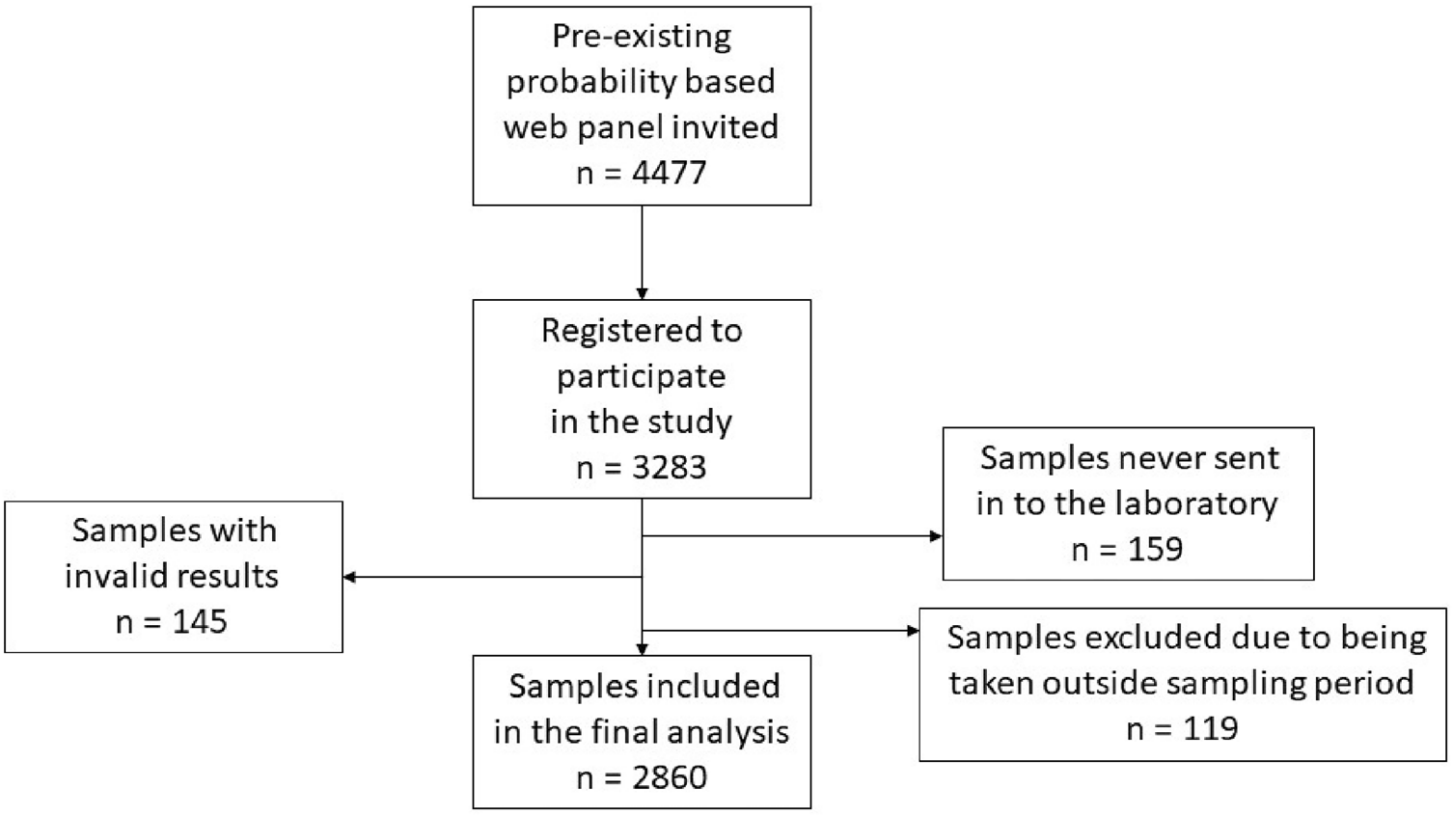
Inclusion of study participants.

Participants were recruited from a national probability-based web panel regularly used for health-related questionnaires at the Public Health Agency of Sweden^3^. Among the 4477 registered individuals in the panel, informed consent for participation in the study was received from 3283 individuals. Participation by minors less than 16 years of age was by informed consent of the respective caretaker and minors 16–17 years of age by informed consent by both themselves and respective caretaker. At-home sampling on dried blood spot (DBS) cassettes was used to collect capillary blood (Capitainer^®^, Solna, Sweden)^4^. Sampling was conducted between April 26 and May 9, 2021. Capillary blood in DBS cassettes was returned to the laboratory and processed for analysis of anti-Spike IgG in an *in-house* SARS-CoV-2 ELISA^5^. Results of the serological analysis were communicated to the participants electronically and by mail. Of the 3283 at-home sampling kits sent out for the study, 423 were either not returned to the laboratory, did not have sufficient amount of capillary blood in them, or were taken outside of the sampling period (Fig. 1). Information about COVID-19 vaccination status and previous confirmed SARS-CoV-2 infection of the participants was collected from the national vaccination registry and the national mandatory notifications system of communicable diseases (SmiNet). Demographics of the participants are summarised in Table 1.

**Table 1:** Distribution of participants, vaccination status, and diagnosed cases, in total and by age group and sex. Sweden April 26–May 9, 2021 (n = 2860).

	Total (n)	Proportion (%)	Proportion vaccinated with at least one dose (%)	Proportion vaccinated with two doses (%)	Proportion diagnosed with COVID-19 (%)
Total	2860	100	34.4	6.4	10.6
0–10 years	170	5.9	0.0	0.0	7.1
11–19 years	219	7.7	0.0	0.0	14.6
20–64 years	1619	56.6	12.8	4.1	13.8
65+ years	852	29.8	91.1	13.5	4.3
Female	1576	55.1	35.4	7.3	11.5
Male	1284	44.9	33.2	5.2	9.5

### Seroprevalence in the population of Sweden

The seroprevalence was calculated as the weighted proportion and was corrected for the laboratory method with the Rogan–Gladen method^6,7^. The weights were based on the sampling weights which were adjusted for the survey non-response with logistic regression and calibrated with the GREG estimator to the population of Sweden with regards to age, sex, education, and geographical region^8^. Estimations are shown with the 95% confidence interval (CI) using the Clopper–Pearson method. Calculations were made in R v.4.0.2. with survey package v.4.0. The seroprevalence of the study population (n = 2860) can be seen in Table 2.

**Table 2:** Weighted seroprevalence with 95% confidence interval in the total study population (n = 2860) and among non-vaccinated (n = 1876), in total and by age group and sex. Sweden April 26–May 9, 2021.

	Proportion with SARS-CoV-2 IgG antibodies			
	Total study population (n = 2860)		Non-vaccinated (n = 1876)	
	%	95% CI	%	95% CI
Total	32.6	(30.3–34.9)	20.1	(17.6–22.8)
0–10 years	20.5	(12.0–31.4)	20.5	(12.0–31.4)
11–19 years	30.5	(23.6–38.2)	30.5	(23.6–38.2)
20–64 years	24.8	(22.0–27.8)	18.0	(15.3–20.9)
65 + years	62.2^a^	(58.6–65.8)	18.1	(9.5–29.6)
Female	34.7	(31.7–37.9)	19.8	(16.6–23.3)
Male	30.4	(27.0–34.0)	20.4	(16.6–24.6)

^a^Seroprevalence is higher in this age group compared to the other age groups (p < 0.001).

Differences between age groups and sex were tested by a weighted logistic regression where a p-value < 0.05 was considered significant. There was a significantly higher proportion of individuals with SARS-CoV-2 antibodies in the age group 65 years and older compared to the other age groups (p < 0.001). No statistical difference in the antibody prevalence by sex was found.

In total, 141 participants of the study population were vaccinated with two doses at least two weeks prior to sampling, and all of these had detectable levels of antibodies against SARS-CoV-2.

### Seroprevalence among non-vaccinated individuals

Table 2 also shows seroprevalence among non-vaccinated individuals (n = 1876) where the highest proportion of individuals with antibodies was detected in the age group 11–19 years (30.5%; 95% CI 23.6–38.2). Seroprevalence in the other age groups ranged from 18.0 to 20.5%. The seroprevalence levels were similar between sexes.

Among non-vaccinated participants with previous laboratory-confirmed SARS-CoV-2 exposure (n = 245), the proportion that had detectable levels of antibodies varied with the time since infection (Table 3). These estimations were adjusted with the Rogan–Gladen method but unweighted^7,9^. The highest proportion was seen among individuals most recently infected (15–90 days before sampling) and was 97.8% (95% CI 92.1–100). In the group that had been infected over 6 months before sampling, 84.4% (95% CI 68.3–94.5) still had detectable levels of antibodies.

**Table 3:** Proportion of non-vaccinated participants previously diagnosed with COVID-19 with detectable antibodies against SARS-CoV-2 divided by the number of days after diagnosis. Sweden April 26–May 9, 2021 (n = 245).

Days since laboratory confirmation of COVID-19 infection	Proportion with SARS-CoV-2 IgG antibodies	
	(%)	95% CI
15–90 days (n = 99)	97.8	(92.1–100)
91–180 days (n = 109)	88.7	(81.0–94.2)
> 181 (n = 37)	84.4	(68.3–94.5)

### Ethics approval

Approval (registration number 2020-07029) was obtained from the Swedish Ethical Review Authority, Uppsala, Sweden. All experiments were performed in accordance with relevant guidelines and regulations (The Act concerning the Ethical Review of Research Involving Humans (Law 2003:460) and ISO/IEC 17025).

### Consent to participate

Informed consent was obtained from all individual participants included in the study. Participation by minors was by informed consent of the respective caretaker.

## Discussion

The results of this point prevalence study suggest that in total 32.6% (95% CI 30.3–34.9) of the population in Sweden had detectable levels of antibodies against SARS-CoV-2 between April 26 and May 9, 2021. This seroprevalence is between previous estimates at national level from March 2021 where 22.4% of blood-donors and 20.7% of outpatients had detectable levels of antibodies and later estimates from the end of May and beginning of June where 51.9% of blood-donors and 52.2% of outpatients had detectable levels^10^. A limitation of the study was that the used method could not distinguish between antibody response induced by a SARS-CoV-2 infection and that induced by vaccination. Due to the higher vaccination coverage in those 65 years and older, the proportion of this group with positive antibody response was higher (62.2%; 95% CI 58.6–65.8, p < 0.001) compared to the other age groups. Among non-vaccinated individuals, those 65+ years had similar antibody levels compared to those 20–64 years and 0–10 years, demonstrating that the higher seroprevalence in the age group 65 + years in the total study population was probably due to vaccination. However, when compared to previous seroprevalence data from Sweden, the proportion of non-vaccinated participants over 65 years with antibodies was still higher in this study^10–13^. It should be noted, though, that there were few participants in this group and thus there is large uncertainty in this estimate. Therefore, in this study the seroprevalence results due to previous infection in the age group 65+ should be interpreted with caution. Another limitation of the study was that it is not possible to verify that the correct individual was sampled as the participants conducted the sampling at home without supervision. However, we consider the risk of receiving sample from the wrong person to be very low as the participants are recruited from a panel that are regularly used for responding to health-related questionnaires and are known for being compliant to instructions given.

Although a correlation between protective immunity and antibody levels has not been determined, waning antibody levels in the group with previous laboratory-confirmed SARS-CoV-2 exposure was indicated over time which is in line with what has been described in other studies and could indicate waning immunity^14–16^. In the group with laboratory-confirmed SARS-CoV-2 exposure within 15–90 days before blood sampling, only 2.2% did not have detectable antibody levels as compared to 15.6% in the group with previous laboratory-confirmed SARS-CoV-2 exposure more than 181 days earlier.

## Conclusions

The study showed that, in total, 32.6% of the population had detectable levels of antibodies, and among non-vaccinated, 20.1% had detectable levels of antibodies in late April and the beginning of May 2021. The antibody response among those having had COVID-19 seems to wane over time with a decreasing proportion of cases with persisting antibody levels with time passed since infection.

## Data Availability

The datasets generated and analysed during the current study are not publicly available due to existing general data protection rules but are available from the corresponding author on reasonable request.
